# Supplementary material for: Energy consumption forecasting for oil and coal in China based on hybrid deep learning
Source: PLoS One. 2025 Jan 6;20(1):e0313856. doi: 10.1371/journal.pone.0313856 (PMC11703084; doi:10.1371/journal.pone.0313856)
Supplement: S1 Appendix — (DOCX) [file pone.0313856.s001.docx]

**Appendix A**

We extracted the experimental data for a time period from 1999 to 2021 with four indicators and 35 features. The four indicators are Production of oil, Import of oil, Production of coal, Import of coal. The 35 features are Natural gas generation, Electricity generation, Total construction industry output, Total fishery output, Total livestock output, Total agricultural output, Total forestry output, Total profits of large-scale industrial enterprises, Number of newly established foreign-invested enterprises, Actual utilized foreign investment amount, Soybean generation, Corn yield, Wheat yield, Rice yield, Soybean import volume, Barley import volume, Corn import volume, Wheat import volume, Rice import volume, Number of graduates from regular higher education institutions, Number of admissions to regular higher education institutions, Number of book publications, Research and development (R&D) expenditure growth rate, Number of residents under minimum living guarantee, Number of participants in maternity insurance, Number of participants in work-related injury insurance, Number of participants in unemployment insurance, Number of units in social service institutions, Employment rate, Labor force, Natural population growth rate of permanent residents, Birth rate of permanent residents, Index of resident consumption level, Engel coefficient of residents, Research and development (R&D) expenditure.

**Appendix B**

We have put some contents cited by the paper here.

Table B1. The results of average correlation coefficient.

| Features/Indicators | Production of oil | Import of oil | Production of coal | Import of coal |
| --- | --- | --- | --- | --- |
| Import of oil | 0.66 | 1.00 | 0.83 | 0.89 |
| Import of coal | 0.76 | 0.89 | 0.93 | 1.00 |
| Natural gas generation | 0.69 | 0.99 | 0.85 | 0.91 |
| Production of oil | 1.00 | 0.66 | 0.85 | 0.76 |
| Production of coal | 0.85 | 0.83 | 1.00 | 0.93 |
| Electricity generation | 0.71 | 0.99 | 0.86 | 0.92 |
| Total construction industry output | 0.69 | 0.99 | 0.85 | 0.92 |
| Total fishery output | 0.70 | 0.98 | 0.85 | 0.91 |
| Total livestock output | 0.73 | 0.97 | 0.85 | 0.89 |
| Total agricultural output | 0.69 | 0.98 | 0.85 | 0.91 |
| Total forestry output | 0.69 | 0.99 | 0.85 | 0.91 |
| Total profits of large-scale industrial enterprises | 0.77 | 0.91 | 0.85 | 0.88 |
| Number of newly established foreign-invested enterprises | -0.12 | 0.28 | 0.09 | 0.13 |
| Actual utilized foreign investment amount | 0.74 | 0.96 | 0.87 | 0.89 |
| Soybean generation | -0.36 | 0.09 | -0.12 | -0.08 |
| Corn yield | 0.76 | 0.91 | 0.83 | 0.86 |
| Wheat yield | 0.65 | 0.88 | 0.77 | 0.81 |
| Rice yield | 0.63 | 0.85 | 0.71 | 0.76 |
| Soybean import volume | 0.71 | 0.97 | 0.84 | 0.89 |
| Barley import volume | 0.41 | 0.68 | 0.51 | 0.57 |
| Corn import volume | 0.54 | 0.76 | 0.71 | 0.73 |
| Wheat import volume | 0.38 | 0.61 | 0.55 | 0.61 |
| Rice import volume | 0.59 | 0.82 | 0.68 | 0.76 |
| Number of graduates from regular higher education institutions | 0.74 | 0.97 | 0.88 | 0.91 |
| Number of admissions to regular higher education institutions | 0.72 | 0.98 | 0.87 | 0.90 |
| Number of book publications | 0.71 | 0.96 | 0.84 | 0.90 |
| Research and development (R&D) expenditure growth rate | -0.54 | -0.67 | -0.56 | -0.63 |
| Number of residents under minimum living guarantee | 0.81 | 0.43 | 0.68 | 0.56 |
| Number of participants in maternity insurance | 0.71 | 0.98 | 0.87 | 0.92 |
| Number of participants in work-related injury insurance | 0.72 | 0.98 | 0.87 | 0.91 |
| Number of participants in unemployment insurance | 0.66 | 0.99 | 0.82 | 0.89 |
| Number of units in social service institutions | 0.66 | 0.96 | 0.81 | 0.91 |
| Employment rate | -0.72 | -0.98 | -0.87 | -0.90 |
| Labor force | 0.89 | 0.78 | 0.83 | 0.82 |
| Natural population growth rate of permanent residents | -0.30 | -0.57 | -0.52 | -0.48 |
| Birth rate of permanent residents | -0.18 | -0.52 | -0.37 | -0.37 |
| Index of resident consumption level | 0.68 | 0.99 | 0.84 | 0.94 |
| Engel coefficient of residents | -0.70 | -0.92 | -0.82 | -0.86 |
| Research and development (R&D) expenditure. | 0.66 | 0.32 | 0.83 | 0.90 |

Table B2. The results of Pearson correlation coefficient.

| Features/Indicators | Production of oil | Import of oil | Production of coal | Import of coal |
| --- | --- | --- | --- | --- |
| Import of oil | 0.65 | 1 | 0.86 | 0.91 |
| Import of coal | 0.79 | 0.91 | 0.92 | 1 |
| Natural gas generation | 0.69 | 0.99 | 0.88 | 0.92 |
| Production of oil | 1 | 0.65 | 0.93 | 0.79 |
| Production of coal | 0.93 | 0.86 | 1 | 0.92 |
| Electricity generation | 0.74 | 0.99 | 0.91 | 0.94 |
| Total construction industry output | 0.68 | 0.99 | 0.86 | 0.94 |
| Total fishery output | 0.72 | 0.98 | 0.88 | 0.95 |
| Total livestock output | 0.78 | 0.97 | 0.93 | 0.94 |
| Total agricultural output | 0.71 | 0.98 | 0.89 | 0.94 |
| Total forestry output | 0.68 | 0.99 | 0.87 | 0.93 |
| Total profits of large-scale industrial enterprises | 0.84 | 0.92 | 0.95 | 0.95 |
| Number of newly established foreign-invested enterprises | -0.08 | 0.37 | 0.15 | 0.12 |
| Actual utilized foreign investment amount | 0.83 | 0.94 | 0.95 | 0.92 |
| Soybean generation | -0.37 | 0.22 | -0.11 | -0.07 |
| Corn yield | 0.83 | 0.93 | 0.93 | 0.96 |
| Wheat yield | 0.73 | 0.89 | 0.85 | 0.89 |
| Rice yield | 0.71 | 0.93 | 0.81 | 0.87 |
| Soybean import volume | 0.75 | 0.98 | 0.9 | 0.93 |
| Barley import volume | 0.44 | 0.8 | 0.57 | 0.67 |
| Corn import volume | 0.36 | 0.67 | 0.53 | 0.59 |
| Wheat import volume | 0.37 | 0.66 | 0.52 | 0.62 |
| Rice import volume | 0.6 | 0.87 | 0.73 | 0.85 |
| Number of graduates from regular higher education institutions | 0.85 | 0.94 | 0.97 | 0.92 |
| Number of admissions to regular higher education institutions | 0.78 | 0.95 | 0.94 | 0.88 |
| Number of book publications | 0.79 | 0.96 | 0.92 | 0.96 |
| Research and development (R&D) expenditure growth rate | -0.57 | -0.77 | -0.65 | -0.77 |
| Number of residents under minimum living guarantee | 0.9 | 0.44 | 0.8 | 0.6 |
| Number of participants in maternity insurance | 0.76 | 0.98 | 0.92 | 0.96 |
| Number of participants in work-related injury insurance | 0.8 | 0.97 | 0.95 | 0.95 |
| Number of participants in unemployment insurance | 0.65 | 0.99 | 0.84 | 0.92 |
| Number of units in social service institutions | 0.66 | 0.97 | 0.82 | 0.92 |
| Employment rate | -0.79 | -0.96 | -0.94 | -0.91 |
| Labor force | 0.95 | 0.8 | 0.96 | 0.87 |
| Natural population growth rate of permanent residents | -0.33 | -0,73 | -0.56 | -0.49 |
| Birth rate of permanent residents | -0.2 | -0.65 | -0.43 | -0.38 |
| Index of resident consumption level | 0.66 | 0.99 | 0.85 | 0.92 |
| Engel coefficient of residents | -0.81 | -0.91 | -0.93 | -0.9 |
| Research and development (R&D) expenditure. | 0.6 | 0.98 | 0.82 | 0.9 |

Table B3. The results of Spearman correlation coefficient.

| Features/Indicators | Production of oil | Import of oil | Production of coal | Import of coal |
| --- | --- | --- | --- | --- |
| Import of oil | 0.74 | 1 | 0.88 | 0.93 |
| Import of coal | 0.82 | 0.93 | 0.97 | 1 |
| Natural gas generation | 0.75 | 1 | 0.89 | 0.94 |
| Production of oil | 1 | 0.74 | 0.88 | 0.82 |
| Production of coal | 0.88 | 0.88 | 1 | 0.97 |
| Electricity generation | 0.75 | 1 | 0.89 | 0.94 |
| Total construction industry output | 0.75 | 1 | 0.89 | 0.94 |
| Total fishery output | 0.75 | 1 | 0.89 | 0.93 |
| Total livestock output | 0.76 | 0.99 | 0.88 | 0.93 |
| Total agricultural output | 0.75 | 0.99 | 0.89 | 0.93 |
| Total forestry output | 0.75 | 1.00 | 0.89 | 0.94 |
| Total profits of large-scale industrial enterprises | 0.79 | 0.95 | 0.86 | 0.91 |
| Number of newly established foreign-invested enterprises | -0.14 | 0.28 | 0.09 | 0.15 |
| Actual utilized foreign investment amount | 0.75 | 0.99 | 0.88 | 0.92 |
| Soybean generation | -0.41 | 0.04 | -0.14 | -0.09 |
| Corn yield | 0.80 | 0.95 | 0.86 | 0.89 |
| Wheat yield | 0.70 | 0.93 | 0.82 | 0.86 |
| Rice yield | 0.68 | 0.88 | 0.76 | 0.79 |
| Soybean import volume | 0.75 | 0.99 | 0.88 | 0.93 |
| Barley import volume | 0.49 | 0.72 | 0.59 | 0.64 |
| Corn import volume | 0.74 | 0.88 | 0.88 | 0.88 |
| Wheat import volume | 0.47 | 0.66 | 0.66 | 0.68 |
| Rice import volume | 0.68 | 0.89 | 0.77 | 0.82 |
| Number of graduates from regular higher education institutions | 0.75 | 1 | 0.89 | 0.94 |
| Number of admissions to regular higher education institutions | 0.75 | 1 | 0.89 | 0.94 |
| Number of book publications | 0.74 | 0.98 | 0.86 | 0.91 |
| Research and development (R&D) expenditure growth rate | -0.62 | -0.73 | -0.63 | -0.69 |
| Number of residents under minimum living guarantee | 0.85 | 0.52 | 0.69 | 0.62 |
| Number of participants in maternity insurance | 0.75 | 0.98 | 0.89 | 0.94 |
| Number of participants in work-related injury insurance | 0.75 | 1 | 0.89 | 0.93 |
| Number of participants in unemployment insurance | 0.74 | 1 | 0.88 | 0.93 |
| Number of units in social service institutions | 0.74 | 0.98 | 0.88 | 0.92 |
| Employment rate | -0.75 | -1 | -0.89 | -0.93 |
| Labor force | 0.92 | 0.83 | 0.84 | 0.86 |
| Natural population growth rate of permanent residents | -0.29 | -0.62 | -0.46 | -0.50 |
| Birth rate of permanent residents | -0.16 | -0.50 | -0.34 | -0.38 |
| Index of resident consumption level | 0.75 | 0.99 | 0.89 | 0.93 |
| Engel coefficient of residents | -0.72 | -0.97 | -0.84 | -0.9 |
| Research and development (R&D) expenditure. | 0.75 | -1 | 0.89 | 0.94 |

Table B4. The results of Kendall correlation coefficient.

| Features/Indicators | Production of oil | Import of oil | Production of coal | Import of coal |
| --- | --- | --- | --- | --- |
| Import of oil | 0.60 | 1 | 0.76 | 0.84 |
| Import of coal | 0.68 | 0.84 | 0.89 | 1 |
| Natural gas generation | 0.63 | 0.98 | 0.79 | 0.87 |
| Production of oil | 1 | 0.60 | 0.75 | 0.68 |
| Production of coal | 0.75 | 0.76 | 1 | 0.89 |
| Electricity generation | 0.63 | 0.98 | 0.79 | 0.87 |
| Total construction industry output | 0.63 | 0.98 | 0.79 | 0.87 |
| Total fishery output | 0.64 | 0.97 | 0.78 | 0.86 |
| Total livestock output | 0.64 | 0.94 | 0.73 | 0.81 |
| Total agricultural output | 0.61 | 0.96 | 0.77 | 0.85 |
| Total forestry output | 0.63 | 0.98 | 0.79 | 0.87 |
| Total profits of large-scale industrial enterprises | 0.67 | 0.87 | 0.73 | 0.79 |
| Number of newly established foreign-invested enterprises | -0.13 | 0.2 | 0.04 | 0.11 |
| Actual utilized foreign investment amount | 0.63 | 0.94 | 0.77 | 0.83 |
| Soybean generation | -0.3 | 0 | -0.11 | -0.09 |
| Corn yield | 0.66 | 0.85 | 0.71 | 0.74 |
| Wheat yield | 0.52 | 0.83 | 0.63 | 0.69 |
| Rice yield | 0.49 | 0.75 | 0.57 | 0.61 |
| Soybean import volume | 0.63 | 0.94 | 0.74 | 0.82 |
| Barley import volume | 0.3 | 0.52 | 0.38 | 0.41 |
| Corn import volume | 0.53 | 0.72 | 0.72 | 0.72 |
| Wheat import volume | 0.29 | 0.51 | 0.46 | 0.53 |
| Rice import volume | 0.5 | 0.71 | 0.55 | 0.61 |
| Number of graduates from regular higher education institutions | 0.63 | 0.98 | 0.79 | 0.87 |
| Number of admissions to regular higher education institutions | 0.63 | 0.98 | 0.79 | 0.87 |
| Number of book publications | 0.61 | 0.93 | 0.74 | 0.82 |
| Research and development (R&D) expenditure growth rate | -0.44 | -0.5 | -0.41 | -0.44 |
| Number of residents under minimum living guarantee | 0.67 | 0.32 | 0.54 | 0.45 |
| Number of participants in maternity insurance | 0.63 | 0.98 | 0.79 | 0.87 |
| Number of participants in work-related injury insurance | 0.62 | 0.98 | 0.78 | 0.86 |
| Number of participants in unemployment insurance | 0.6 | 0.98 | 0.75 | 0.83 |
| Number of units in social service institutions | 0.58 | 0.93 | 0.74 | 0.9 |
| Employment rate | -0.62 | -0.98 | -0.78 | -0.86 |
| Labor force | 0.81 | 0.7 | 0.7 | 0.72 |
| Natural population growth rate of permanent residents | -0.28 | -0.52 | -0.52 | -0.44 |
| Birth rate of permanent residents | -0.19 | -0.42 | -0.33 | -0.36 |
| Index of resident consumption level | 0.62 | 0.98 | 0.78 | 0.96 |
| Engel coefficient of residents | -0.57 | -0.88 | -0.69 | -0.79 |
| Research and development (R&D) expenditure. | 0.63 | 0.98 | 0.79 | 0.87 |

Fig.B1. The situations of the real values and the corresponding forecasted values with our proposed ensemble approach.

Fig.B2. Hyperparameter analysis of the single LSTM model. Four rows represent the four indicators of production of oil, production of coal, import of oil, and import of coal, respectively.

Fig.B3. The adaptive ensembling weights of our approach. Five rows represent 2017-2021, respectively, where four figures of each row are the results of production of oil, production of coal, import of oil, and import of coal, respectively.

Fig.B4. The convergence curves of all the involved models. Five rows represent 2017-2021, respectively, where four figures of each row are the results of production of oil, production of coal, import of oil, and import of coal, respectively.
