# Supplementary material for: Energy consumption forecasting for oil and coal in China based on hybrid deep learning
Source: PLoS One. 2025 Jan 6;20(1):e0313856. doi: 10.1371/journal.pone.0313856 (PMC11703084; doi:10.1371/journal.pone.0313856)
Supplement: Attachment — Submitted filename: Response.doc [file pone.0313856.s003.doc]

**Author’s Detailed Responses to Reviewers’ Comments for Paper
Energy Consumption Forecasting for Oil and Coal in China Based on Hybrid Deep Learning (PONE-D-24-27898R1)**

***Associate Editor***

*Comments: The Y axis of figure 6 (previously figure 7 in the original manuscript) as contained in the reviewer’s feedback comment was still not corrected. Should be corrected before publication!*

**Response**: The authors highly appreciate the AE’s recommendation and comments. We have revised the Y axis of figure 6. Please note that all the modifications we’ve made are colored blue in the revised manuscript. Thank you very much for handling this paper.

***Reviewer #*1**

RQ1. *The authors met all of my comments! I believe this is now a novel and interesting study and the paper reflects that!*

**Response:** The authors highly appreciate the Reviewer for your recommendation.

***Reviewer* 2**

*The authors have implemented majority of the comments/concerns raised in the first manuscript. This article should be accepted for final publication, subject to the following minor revisions.*

**Response:** The authors highly appreciate the Reviewer’s encouraging comments. The manuscript has been carefully revised with care according to your raised minor revisions. Detailed responses are provided below.

RQ1. *Table 14. The case of our approach loses the comparison is row of ‘2020’ in “Production of coal, not “Import of oil”*

**Response:** The authors highly appreciate the Reviewer for pointing this issue out. We have fixed this issue. Please review the revised manuscript.

RQ2. *The authors have didn’t change the y-axis range in Figure 6 (previously figure 7 in the original manuscript) as contained in the reviewer’s feedback comment. Should be corrected before publication!*

**Response:** The authors highly appreciate the Reviewer for pointing this issue out. We have changed the y-axis range in Figure. Please review the revised manuscript.

RQ3. *Mix of citation style in Introduction paragraph 2. E.g Tong et al(date?). and Rao et al(date?) is used together with numbered citations (e.g [1], [2], [3],…etc). If this is acceptable to the journal, fine. Otherwise, the authors should correct/unify these before final publication*

**Response:** The authors highly appreciate the Reviewer for pointing this issue out. We have revised this issue in Introduction. Please review the revised manuscript.

***The authors would like to thank all editors and reviewers for their insightful and constructive comments that have greatly helped us improve our study and manuscript.***
